# Evaluation of a Novel Quantitative Multiparametric MR Sequence for Radiation Therapy Treatment Response Assessment

**Published:** 2025-03-28

**Authors:** Yuhao Yan, R. Adam Bayliss, Florian Wiesinger, Jose de Arcos Rodriguez, Adam R. Burr, Andrew M. Baschnagel, Brett A. Morris, Carri K. Glide-Hurst

**Affiliations:** * Department of Human Oncology, University of Wisconsin-Madison, Madison, WI; † Department of Medical Physics, University of Wisconsin-Madison, Madison, WI; ‡ GE HealthCare, Munich, Germany; § GE HealthCare, Little Chalfont, United Kingdom

## Abstract

**Background:**

Multi-parametric MRI has shown great promise to rapidly derive multiple quantitative imaging biomarkers for treatment response assessment.

**Purpose:**

To evaluate a novel Deep-Learning-enhanced MUlti-PArametric MR sequence (DL-MUPA) for treatment response assessment for brain metastases patients treated with stereotactic radiosurgery (SRS) and head-and-neck (HnN) cancer patients undergoing conventionally fractionation adaptive radiation therapy.

**Methods:**

DL-MUPA derives quantitative T1 and T2 relaxation time maps from a single 4–6-minute scan denoised via DL method using least-squares dictionary fitting. Longitudinal phantom benchmarking was performed on a NIST-ISMRM phantom over one year. In patients, longitudinal DL-MUPA data were acquired on a 1.5T MR-simulator, including pre-treatment (PreTx) and every ~3 months after SRS (PostTx) in brain, and PreTx, mid-treatment and 3 months PostTx in HnN. Delta analysis was performed calculating changes of mean T1 and T2 values within gross tumor volumes (GTVs), residual disease (RD, HnN), parotids, and submandibular glands (HnN) for treatment response assessment. Uninvolved normal tissues (normal appearing white matter in brain, masseter in HnN) were evaluated to quantify within-subject repeatability.

**Results:**

Phantom benchmarking revealed excellent inter-session repeatability (coefficient of variance <0.9% for T1, <6.6% for T2), suggesting reliability for longitudinal studies once systematic biases are adjusted. Uninvolved normal tissue suggested acceptable within-subject repeatability (brain |ΔT1_mean_|<36ms/5.0%, |ΔT2_mean_|<2ms/5.0%, HnN |ΔT1_mean_|<69ms/7.0%, |ΔT2_mean_|<4ms/17.8% due to low T2). In brain, remarkable changes were noted in resolved metastasis (4-month PostTx ΔT1_mean_=155ms/13.7%) and necrotic settings (ΔT1_mean_=214–502ms/17.6–39.7%, ΔT2_mean_=7–41ms/8.7–41.4%, 6-month to 3-month PostTx). In HnN, two base of tongue tumors exhibited T2 enhancement (PostTx GTV ΔT2_mean_>7ms/12.8%, RD ΔT2_mean_>10ms/18.1%). A case with nodal disease resolved PostTx (GTV ΔT1_mean_=-541ms/-39.5%, ΔT2_mean_=-24ms/-32.7%, RD ΔT1_mean_=-400ms/-29.2%, ΔT2_mean_=-25ms/-35.3%). Enhancement was found in involved parotids (PostTx ΔT1_mean_>82ms/12.4%, ΔT2_mean_>6ms/13.4%) and submandibular glands (PostTx ΔT1_mean_>135ms/14.6%, ΔT2_mean_>17ms/34.5%) while the uninvolved organs remained stable.

**Conclusions:**

Preliminary results suggest promise of DL-MUPA for treatment response assessment and highlight potential endpoints for functional sparing.

## Introduction

With its excellent soft tissue contrast and increased availability of MR-simulators (MR-SIMs) sited in Radiation Oncology, MRI has played a vital role in radiation therapy (RT) including treatment response assessment^1^ through the application of quantitative MR imaging (QMRI) biomarkers to quantify the changes in physical and functional response to support clinical decision-making.^2,3^

In the brain, MRIs including post-Gadolinium T1-weighted and FLAIR T2-weighted MRIs are routinely acquired for management of glioblastoma and metastases while challenges remain in differentiating between radiation necrosis, tumor progression, or recurrence using conventional qualitative MRIs. ^4,5^ Diffusion-weighted imaging and perfusion-weighted imaging have been heavily explored to measure QMRI parameters including apparent Diffusion coefficient (ADC) and relative cerebral blood volume, respectively, for glioblastoma and metastases radiation treatment response assessment.^6,7^ Ding et al. prospectively assessed 56 brain metastases patients treated by Gamma Knife radiosurgery who showed new enhancing mass ≥5-months post-treatment and found the difference between quantitative T1 relaxation time 5-minute and 60-minute post contrast administration sensitive to tumor recurrence.^8^

In Head and Neck (HnN), to accommodate drastic tumor and anatomical changes that occur during the treatment course, recent advancements of adaptive radiation therapy (ART) strategies have demonstrated dosimetric benefits of organs at risk (OAR) sparing and potential clinical benefits in terms of survival and toxicity.^9^ ART allows mid-RT MR acquisitions and subsequent longitudinal QMRI biomarker analysis which can potentially enhance response assessment and guide the adaptation.^10^ Mohamed et al. prospectively evaluated 81 HnN cancer patients treated with conventionally fractionated RT and identified changes of mean ADC values in primary tumor volume greater than 7% at the time of mid-RT (~18 RT fractions) compared to pre-RT as a statistically significant QMRI biomarker associated with better local control and recurrence-free survival.^11^ Bonate et al. prospectively analyzed 15 HnN squamous cell cancer (SCC) patients treated with hypofractionated ART on a 1.5T MR-linac and found significant longitudinal increase in ADC and T2 values of primary tumor over the cohort.^12^

With the promising application of QMRI in RT response analysis, it is appealing to have quantitative multi-parametric MRI (mpMRI) to provide comprehensive functional information, however routine application of mpMRI is currently limited by the need to acquire multiple MRIs with long acquisition time to achieve clinically acceptable resolution,^13^ particularly for conventional T1 and T2 quantification methods such as variable flip angle (VFA) and inversion recovery (IR). Recently, Nejad-Davarani et al. optimized and implemented a novel mpMRI sequence, STAGE (STrategically Acquired Gradient Echo), on patients with glioblastoma treated on a 0.35T MR-linac with conventional fractionation RT, which acquired quantitative proton density (PD), R2* and T1 maps in 10 minutes. This pilot study observed changes of above QMRI values in primary tumor volume at follow up (2 months) agreeing to findings on diagnostic images.^14^ The technique of MR Fingerprinting (MRF) has recently emerged, which simultaneously derives multiple QMRI values in a single scan, matching unique signal evolution of different tissue under the sequence with continuously varying parameters to a pre-calculated dictionary.^15^ Given the promising efficacy, efforts have been devoted to clinical translation of mpMRI to facilitate ART and response assessment, focusing on the evaluation of accuracy, repeatability and in-vivo feasibility.^16,17^ In the present work, a vendor-developed deep-learning-enhanced multi-parametric MR sequence, ‘DL-MUPA’, was implemented that yields 5 qualitative datasets and derives quantitative T1 and T2 relaxation time maps from a single 4–6-minute scan. Accuracy and repeatability were first assessed in a comprehensive phantom study and results were demonstrated in two patient cohorts: brain metastases patients undergoing stereotactic radiosurgery (SRS) and HnN cancer patients undergoing conventionally fractionated ART.

## Methods

### Deep-Learning-enhanced MUlti-PArametric MR Sequence – DL-MUPA

DL-MUPA (GE Healthcare, software versions 29.1–30.1, Milwaukee, WI) sequence acquires 1 PD, 3 T1 and 1 T2 weighted qualitative 3D images in a single 4–6-minute scan and derives quantitative T1 and T2 relaxation time maps using least-squares, orthogonal matching pursuit^18^ dictionary fitting relative to spoiled gradient-echo (SPGR) simulated signals.^19,20^ The sequence starts with a steady-state PD-w image acquisition using low flip angle (FA, ~1°) Zero TE, followed by a transient-state T1 and T2 magnetization prepared 4-segment Zero TE acquisition (FA=3°) similar to the 3D-QALAS approach, where one acquisition was performed after the T2 preparation, then three acquisitions were performed at different points of the T1 relaxation after the T1 preparation.^21^ Images were reconstructed using a DL-based reconstruction pipeline, which uses a deep convolutional neural network (CNN) to reduce noise, ring artifacts and improve sharpness.^22,23^ The minimal gradient switching intrinsic to Zero TE has been characterized as “silent” that noise is minimized during acquisition.^24^

### Phantom Benchmarking

#### Phantom and Data Acquisition

A NIST-ISMRM quantitative MR phantom (Serial Number 0130, CaliberMRI, Boulder, CO) was scanned to benchmark the DL-MUPA sequence. The phantom contains arrays of 14 sphere vials of NiCl_2_ and MnCl_2_ solutions at different concentrations for T1 and T2, respectively, spanning across T1 values of [22–1741 ms] and T2 values of [7–493 ms] per manufacturer reference, calculated based upon a NIST-provided methodology at 1.5T at 20°C. Following guidance from the manufacturer, two T2 vials (index 1 and 5) were excluded from formal analysis as reported elsewhere.^25,26^

Phantom benchmarking was performed following Quantitative Imaging Biomarker Alliance (QIBA) guidelines.^2,3^ The phantom was scanned with DL-MUPA sequence using a 19-channel brain coil (GE GEM head and neck suit) on a GE 1.5T SIGNA Artist MR which is dedicated to radiation oncology use. A single DL-MUPA scan took 4 min 30 sec with 1.6 mm^3^ acquisition voxel size, 2 averages, 202 mm^3^ FOV covering the whole phantom. To test the accuracy and repeatability of the sequence, 12 separate phantom imaging sessions were acquired over one year with 5 consecutive repeat scans at each scanning session. The phantom was stored in the scan room to maintain thermal equilibration and phantom temperature was measured before and after each session to assess the need for temperature corrections.

#### Image Processing and Quantitative Analysis

An automated MATLAB algorithm was devised to segment the vials on PD-w images as the contrast and image quality provided definite boundaries of the vials. Each vial was measured by the mean intensity of voxels within 5 mm radius to avoid partial volume effects. Physiologically relevant T1 [246–1741 ms] and T2 [42–493 ms] values were assessed. Accuracy was evaluated by bias comparing mean derived values of all acquisitions (12×5) to manufacturer-provided reference, in both absolute value and percentage relevant to the reference. Repeatability was measured by coefficient of variance ($CV=\frac{standard\;Deviation}{Mean}$). For each vial, intrasession CV was calculated over the 5 consecutive acquisitions in a single session, thus rendering 12 intrasession CVs, of which only the range was reported. Mean T1 and T2 of each session was calculated averaging the five acquisitions. Intersession CV was calculated over the 12 session means. Linearity between the derived values against the reference were assessed using least squares linear regression via Scipy package in python.

### In-vivo Feasibility Study

#### Patient Cohort and Data Acquisition

To study the feasibility of using DL-MUPA derived quantitative T1 and T2 maps to assess treatment response *in vivo*, longitudinal DL-MUPA images were prospectively acquired on IRB-approved protocols to enable experimental longitudinal imaging, one for brain metastases patients and one for HnN cancer patients.

The brain metastases patients underwent SRS to 18–21 Gy. Pre-contrast DL-MUPA scans were acquired prior to SRS (PreTx) and at follow-ups every ~3 months after SRS procedure (PostTx). Patients were scanned on the GE 1.5T SIGNA Artist MR with the 19-channel brain coil, identical to phantom benchmarking. DL-MUPA took 4 min 30 sec with 1.5 mm^3^ acquisition voxel size, 192 mm^3^ FOV covering the whole brain, 2 averages. Post-contrast T1 Stealth (repetition time (TR)~9 ms, echo time (TE)~3.5 ms, inversion time (TI)=450 ms, FA=13°) and T2 FLAIR (TR=6000 ms, TE~135 ms, TI~1730 ms, FA=90°, echo train length (ETL)=200) were also acquired at each timepoint. CT simulation was performed on Siemens SOMATOM Definition Edge (120 kV) the same day as PreTx MR acquisition.

The HnN cancer patients underwent conventional fractionation ART to 70–76 Gy with plan adaptation performed after the 10^th^-15^th^ RT fraction to accommodate anatomical changes. Planning objectives included D_mean_<26 Gy for parotids and <39 Gy for submandibular glands.^27,28^Pre-contrast DL-MUPA scans were acquired at the time of MR-SIM prior to treatment (PreTx), 2–3 weeks after RT start (MidTx), and at 3-month follow-up after treatment end (PostTx). Patients were scanned in treatment position with their immobilization masks on the GE 1.5T SIGNA Artist MR with a 32-channel Head and Neck coil (GE AIR Open RT suite) or a 30-channel Head and Neck coil (GE GEM RT Open suite). For HnN, DL-MUPA was acquired in 6 min 30 sec with 1.5×1.5×2 mm^3^ acquisition voxel size, 264 mm^3^ FOV covering from the apex of lung to the middle level of the brain, 1.5 averages. Pre-contrast 2D T2 frFSE (TR~9000 ms, TE~77 ms, ETL=19, FA=160°) and post-contrast T1 LAVA-Flex (TR~6 ms, TE~3 ms, FA=12°) were also acquired at each timepoint. PreTx CT simulation was performed on the Siemens SOMATOM Definition Edge (120 kV) 3.4±3.3 days (range: [0, 10] days) from MR-SIM.

#### Image Processing and Delta Analysis

For each site, clinically approved contours were initially delineated on the treatment planning CTs (TPCTs). Using MIM Maestro v7.2.8 (MIM software, Cleveland, OH), in brain, longitudinal DL-MUPA T1-w images were rigidly co-registered to TPCT using box-based assisted alignment followed by manual adjustments.^14^ GTVs were then rigidly propagated from TPCT to DL-MUPA images. In HnN, longitudinal T2 frFSE images were co-registered to TPCT via multi-modality deformable registration (DIR) followed by manual adjustments locally near the lesion. DL-MUPA T1-w images were rigidly co-registered to T2 frFSE images at corresponding timepoints using localized box-based alignment.^11^ GTVs were propagated to longitudinal T2 frFSE and contours were finalized by a board-certified HnN radiation oncologist. As substantial GTV changes were observed over the treatment course, the MidTx and PostTx Residual Disease (RD) volumes were delineated using information from the T2 frFSE and post-contrast T1 LAVA-Flex following a method defined by Mohamed et al.^11^ The clinically used parotid and submandibular gland volumes were deformably propagated to the other T2 frFSE timepoints and modified to match the underlying anatomy.^29^ All finalized contours were then rigidly propagated from T2 frFSE to DL-MUPA images for quantitative T1 and T2 analysis.

To define uninvolved tissue as a control for longitudinal datasets, in brain, the normal appearing white matter (NAWM)^14^ was derived by subtracting the lesion from white matter automatically segmented on T1-w Stealth and T2-w FLAIR images using SPM12 and LST toolbox.^30,31^ Analysis was then conducted based on the initial timepoint at the central plane of the GTVs, in the uninvolved contralateral or ipsilateral hemisphere, then propagated to the secondary timepoint via rigid registration for longitudinal assessment. For HnN, a single slice of uninvolved contralateral masseter^32^ was manually delineated on PreTx images as a control volume.

To mask out the background, Otsu thresholding^33^ was performed on the DL-MUPA-derived PD-w images. In addition, in HnN, the airway was carefully masked out by implementing seed fill algorithm. For quantitative analysis, mean T1 and T2 values within each region of interest (ROI) were calculated at each timepoint. Delta analysis was performed calculating longitudinal changes of mean T1 and T2 values with respect to the first available timepoint for ROIs including GTV, parotids and submandibular glands for HnN, and uninvolved normal tissue (contralateral NAWM in Brain and masseter in HnN).^14,27,28,32^ In addition, for HnN, major anatomical changes were addressed by isolating the residual disease and comparing mean T1 and T2 values within MidTx/PostTx RD to PreTx GTV (initial disease) via histogram analysis. ^11^ Furthermore, for each patient, within-subject coefficient of variance $wCV=\frac{standard\;Deviation}{Mean}$ was calculated for each ROI across all analyzed timepoints.

## Results

### Phantom Benchmarking

Table 1 summarizes the quantitative results of phantom benchmarking across 12 sessions. Mean derived T1 and T2 values showed systematic bias of ~25–35% compared to manufacturer reference. By contrast, intersession CVs were minimal within 0.9% for all T1 values and within 2.4% for most T2 values except for vial #2 (4.8%) and #9 (6.6%), indicating excellent intersession repeatability. Similarly, minimal intrasession variations were observed (CVs for T1 within 1% and T2 within 4.3%). Intra-session and inter-session temperature variations were negligible (<0.5°C and <1°C, respectively), thus no temperature calibration was deemed necessary.

**Figure 1.**
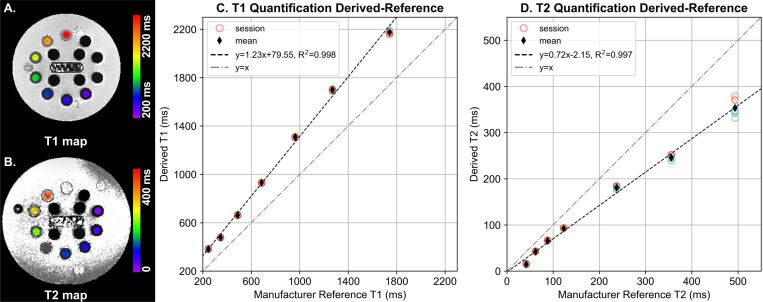
Example (A) T1 and (B) T2 map of corresponding vial array derived on the phantom by DL-MUPA. Derived (C) T1 and (D) T2 values are plotted against manufacturer-provided reference with each circle standing for mean derived value of five acquisitions in a single session and each thin diamond standing for mean derived value over all acquisitions (12×5). Least square linear regression lines were plotted in black dash line and identity lines were plotted in grey dash-dot line.

Figure 1C and 1D summarizes the mean T1 and T2 values in each session as compared to the manufacturer-provided reference data. While linear regression fitting revealed systematic biases, excellent intersession repeatability was observed as characterized by tightly clustered datapoints which overall deviated from the identity line. Strong linear associations between the DL-MUPA derivations and the manufacturer reference were observed for both T1 (R^2^ = 0.998) and T2 (R^2^ = 0.997).

### Brain Metastases Cohort

Five subjects were evaluated for NAWM and exhibited minimal variations with |ΔT1_mean_| < 20 ms (2.9%), ΔT2_mean_ < 2 ms (5.0%) between different timepoints, except for one subject showing marginal change with ΔT1_mean_ of 36 ms (5.0%). wCV of NAWM was 0.3%-3.5% for T1 and 0.8%-3.4% for T2.

**Figure 2.**
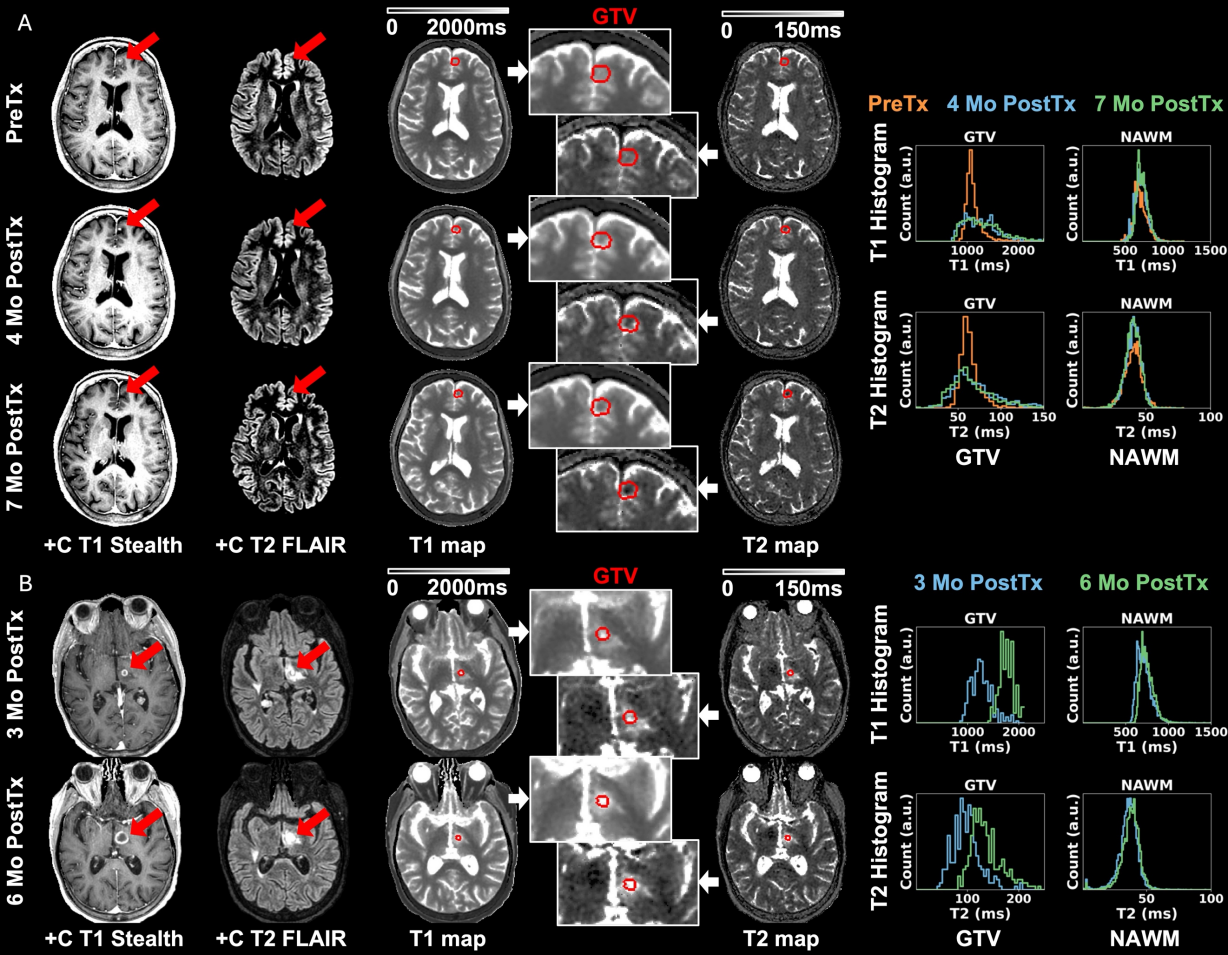
Post-contrast T1 Stealth and T2 FLAIR, longitudinal DL-MUPA derived T1 and T2 maps (global and magnified to metastases), and T1 and T2 histograms for two different brain metastases patients. Normal appearing white matters (NAWM) remained consistent, demonstrating reliability of DL-MUPA in longitudinal studies. Patient A experienced lesion shrinkage at 4-month PostTx leading to T1 and T2 changes, which remained stable 7-month PostTx. T1-w and T2-w images were shown in a narrow window to highlight the changes. Patient B exhibited newly identified lesion necrosis at 6-month PostTx which showed remarkable T1 and T2 enhancement compared to 3-month PostTx.

Table 2 summarizes key results from two patients A-B including longitudinal changes and wCV of T1 and T2 within each ROI, illustrated in detail as follows, respectively. Figure 2 highlights key results of two different patients with brain metastases acquired over a 7-month timeframe. For the patient in Fig 2A, NAWM histograms demonstrated reliable consistency for both T1 and T2 quantification (|ΔT1_mean_ |<2.9%, |ΔT2_mean_|<0.9%). Within the GTV, the 4-month PostTx T1 and T2 maps highlighted an increase in both T1 (ΔT1_mean_=13.7%) and T2 (ΔT2_mean_=17.8%). 7-month PostTx QMRI maps and histograms agreed with 4-month PostTx. Fig 2B summarizes key results of another brain metastases patient whose PreTx DL-MUPA images suffered from motion artifacts thus not included for analysis. NAWM histograms demonstrated close agreement (ΔT1_mean_=5.0%, ΔT2_mean_=5.0%). The left thalamus lesion exhibited central necrosis identified on the 6-month PostTx +C T1 Stealth that corresponded to notable enhancement on T1 and T2 maps (ΔT1_mean_=39.7%, ΔT2_mean_=41.4%) compared to 3-month PostTx. Two other metastases of this patient were also identified as necrosis 6-month PostTx, key results summarized in Appendix Figure S1. The right precentral gyrus lesion (GTV2) demonstrated similar enhancement (ΔT1_mean_=17.6%, ΔT2_mean_=8.7%) while the left middle frontal gyrus lesion (GTV3) showed minimal changes (ΔT1_mean_=-4.6%, ΔT2_mean_=-7.1%).

### HnN Cancer Cohort

Table 3 summarizes key results of three patients I-III including longitudinal changes and wCV of T1 and T2 within each ROI, illustrated in detail in the following paragraphs, respectively.

**Figure 3.**
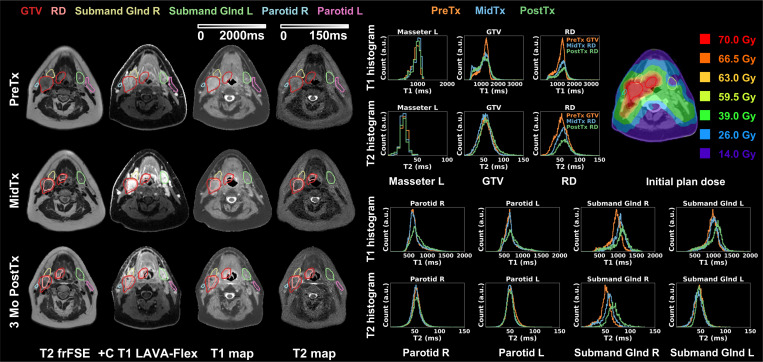
Longitudinal T2 frFSE, post-contrast T1 LAVA-Flex, DL-MUPA derived T1 and T2 maps, quantitative T1 and T2 histograms, and initial plan dose for one Head and Neck cancer patient. Uninvolved masseter remained consistent at all timepoints, demonstrating reliability of DL-MUPA in longitudinal studies. This patient exhibited tumor resolution on both T1 and T2 map and longitudinal changes of involved ipsilateral submandibular gland.

Fig 3 summarizes key results of a patient with Stage I (T1N1) SCC of the right base of tongue. The treatment plan was adapted MidTx due to substantial decrease in tumor size. This patient was imaged with the GE AIR Open RT suite and immobilized at each timepoint. The uninvolved masseter remained stable (|ΔT1_mean_|<5.0%, |ΔT2_mean_|<5.6% across all timepoints). Longitudinal resolution of tumor was reflected on qualitative images and DL-MUPA derived T1 and T2 maps. GTV T1 and T2 histograms showed slight changes while Delta analysis suggests increased T2 PostTx (ΔT2_mean_=12.8%). For the changing residual disease over time, the T1 and T2 histograms skewed toward higher values while Delta analysis revealed stable T1 but increased T2 PostTx (ΔT2_mean_=18.1%). Due to the extensive disease, the ipsilateral submandibular gland received a mean dose of 64 Gy. The T1 and T2 histograms showed substantial shifts toward higher value (PostTx ΔT1_mean_=14.6%, ΔT2_mean_=34.5%). Similarly, the involved ipsilateral parotid showed increasing T1 PostTx (ΔT1_mean_=12.4%) but stable T2. By contrast, despite some changes on T1 histograms, the contralateral submandibular gland with a mean dose of 33 Gy had near-stable T1 (|ΔT1/T2_mean_|<7.3%) and uninvolved parotid had negligible variations (|ΔT1/T2_mean_| <5.6%). The patient reported gradually worsening dry mouth at treatment end and 3-month follow-up (PostTx).

**Figure 4.**
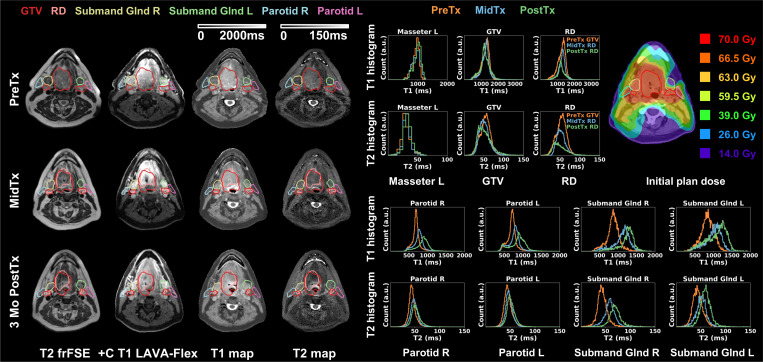
Longitudinal T2 frFSE, post-contrast T1 LAVA-Flex, DL-MUPA derived T1 and T2 maps with corresponding quantitative T1 and T2 histograms, and corresponding dose distribution for a Head and Neck cancer patient. Stable T1 and T2 data across timepoints are evident in the uninvolved masseter demonstrating reliability of DL-MUPA in longitudinal studies. Longitudinal changes in the salivary glands were consistent with clinical response as described in text.

Figure 4 highlights results from a patient with Stage IV (T4N2) SCC of the right base of tongue who exhibited ~10 kg weight loss MidTx necessitating plan adaptation. This patient was imaged with the GE AIR Open RT suite at each timepoint but not immobilized due to claustrophobia. The uninvolved masseter remained stable except for slightly higher T2 variations at PostTx (|ΔT1_mean_|<5.7%, |ΔT2_mean_|<11.8%). Changes of solid tumor around base of tongue were observed especially on T2 map. Within GTV, variations in mean T1 and T2 were negligible (|ΔT1/T2_mean_|<2.5%) despite some histogram changes. RD histogram peaks shifted towards lower values while the mean T1 and T2 showed minimal changes as well (|ΔT1/T2_mean_|<4.4%). The submandibular glands demonstrated substantial changes in quantitative MRI endpoints with PostTx ΔT1_mean_=43.8%, ΔT2_mean_=61.3% and ΔT1_mean_=28.9%, ΔT2_mean_=36.4% for the ipsilateral (receiving mean dose of 69 Gy) and contralateral submandibular glands, respectively. Changes were also observed for the ipsilateral (ΔT1_mean_=26.6%, ΔT2_mean_=18.4%) and contralateral (ΔT1_mean_=22.3%, ΔT2_mean_=13.4%) parotids. The patient reported persistent severe dry mouth throughout and after treatment.

**Figure 5.**
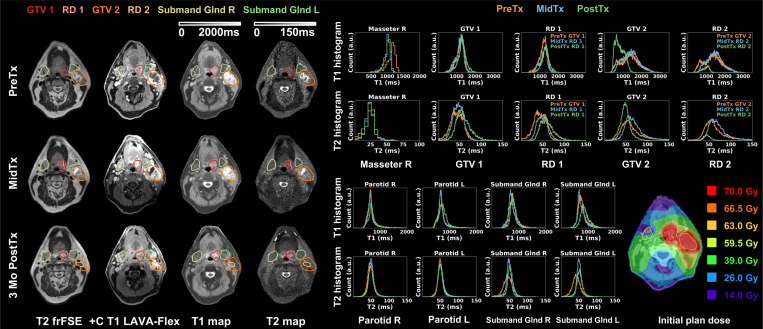
Longitudinal T2 frFSE, post-contrast T1 LAVA-Flex, DL-MUPA derived T1 and T2 maps, quantitative T1 and T2 histograms, and initial plan dose for one Head and Neck cancer patient. Uninvolved masseter showed discrepancy in T1 quantification potentially linked to the use of different coils. This patient exhibited regional changes in base of tongue primary tumor (GTV 1) and resolution of significant nodal metastases (GTV 2) at PostTx. Glands were generally stable. Left submandibular gland T1 histogram showed noticeable PostTx changes. T2 histogram mismatches were found between PreTx and following timepoints for both submandibular glands.

Figure 5 demonstrates results from patient diagnosed with Stage I (T1N1) SCC of the left base of tongue (red) with significant nodal metastases (orange). The patient’s plan was not adapted due to limited changes in anatomy during the treatment course. Because of coil evolution during the study, GE GEM RT Open suite was used PreTx whereas GE AIR Open RT suite was used MidTx and PostTx, all with immobilization. For uninvolved masseter, MidTx and PostTx T1 histograms showed excellent agreement but both deviated from PreTx T1 histogram (|ΔT1_mean_|>10.5%). T2 quantification was consistent instead (|ΔT2_mean_ |<8.1% (2 ms)). The base of tongue tumor showed slight changes with regional enhancement observed on PostTx T1 and T2 maps (GTV ΔT1_mean_=7.6%, ΔT2_mean_=33.4%, RD ΔT1_mean_=9.9%, ΔT2_mean_=36.6%). The significant nodal metastases largely resolved PostTx, leading to substantial changes in QMRI endpoints (GTV ΔT1_mean_=−39.5%, ΔT2_mean_=−32.7%, RD ΔT1_mean_ = −29.2%, ΔT2_mean_=−35.3%). Both parotids remained stable. The involved ipsilateral submandibular gland received mean dose of 64 Gy and exhibited T1 enhancement PostTx (ΔT1_mean_=15.1%) while the uninvolved one showed less substantial change (|ΔT1_mean_|<8.1%). For both submandibular glands, T2 histograms showed similar pattern with agreement between MidTx and PostTx but mismatch from PreTx. The patient reported severe dry mouth at the end of treatment which was resolved PostTx.

Across five subjects analyzed, uninvolved contralateral masseter exhibited higher T1 variations for patients who were imaged with different coils. Within the same subject, comparing timepoints acquired with the same coil (3 patients), |ΔT1_mean_|<69 ms (7.0%) and wCV<3.7%, whereas |ΔT1_mean_|=118–142 ms (10.5%-12.6%) and wCV=6.9%-7.3% comparing timepoints acquired with different coils (2 patients). |ΔT2_mean_| < 4 ms (17.8% due to extremely low absolute T2 value of masseter) and wCV<10.8% with no discrepancy seen when using different coils.

## Discussion

Our work evaluated a novel mpMRI sequence, DL-MUPA, for its feasibility of radiation treatment response assessment. The phantom study revealed excellent repeatability of the sequence although a systematic bias was identified, suggesting reliability in longitudinal studies after the offset is addressed. When the same coil was used, stable longitudinal results were obtained in uninvolved normal tissue in brain and HnN, suggesting promising within-subject repeatability consistent with the literatures.^14,34–37^ Quantitative changes in GTVs corresponded to brain metastases resolution/necrosis and HnN tumor resolution, respectively. T1 and T2 enhancement in involved parotids and submandibular glands coincided with patients’ reported salivary function changes.

Despite systematic bias compared to the phantom reference values, our DL-MUPA phantom benchmarking demonstrated a strong association of both T1 and T2 derivation to the reference (R^2^>0.997) and excellent longitudinal repeatability (intersession CV<0.9% for T1 and <6.6% for T2), suggesting reliability in longitudinal studies. Systematic bias of T1 and T2 quantification is often reported when benchmarking QMRI sequences using the phantom. In physiological ranges, Carr et al.^25^ reported 2.0%-8.5% median bias of T1 and T2 quantification on a 3T MR in a 1-year phantom benchmarking of conventional sequences, and Nejad-Davarani et al.^14^ reported 9.5% mean bias comparing the STAGE T1 quantification against inversion recovery for a 0.35T MR-linac. Nevertheless, excellent longitudinal repeatability and linearity were reported in the above studies (intersession CV <6% and R^2^>0.997 reported in similar T1 and T2 range) ^14,25^, agreeing with our results.

In NAWM in the brain, excellent longitudinal agreement (<5%) in T1 and T2 values as well as low within-subject CV (<3.5%) were observed that were comparable to the literature (CV=1.5%-12.8% for T1, ~5% for T2)^14,34,35^ demonstrating the reliability of the sequence. For absolute NAWM quantification, our study showed considerable T1 overestimation and T2 underestimation compared to literature,^38^ consistent with the bias of higher T1 and lower T2 observed in phantom. For metastatic brain tumor response, a resolved brain metastases demonstrated T1 and T2 enhancement (ΔT1_mean_=13.7%, ΔT2_mean_=17.8%) comparing 4-month PostTx to PreTx scans while two necrotic brain metastases also exhibited T1 and T2 enhancement (ΔT1_mean_=17.6%-39.7%, ΔT2_mean_=8.7%-41.4%). Limited data are available for comparison in the literature. Konar et al.^39^ utilized synthetic MRI (MAGnetic resonance Image Compilation) derived T1 and T2 maps to characterize brain metastases on a 3T MR. Each patient was imaged only once, either before or after the treatment, thus no same-subject comparison was available. They reported increases in both T1 (Δ=18.4%) and T2 (Δ=14.0%) comparing treated to untreated brain metastases

In HnN, uninvolved contralateral masseter showed good T1 consistency when imaged with the same coil (|D|<7.0%, CV<3.7%), while higher variations up were found when imaged with different coils (|D|>10.5%, CV>6.9%). T2 quantification showed no discrepancy when using different coils (CV=3.4%-10.8%). While data is limited, literatures have reported repeatability of other QMRI in HnN including T1rho CV=3.3%-6.5% and ADC CV=5.7%-10.0% for normal tissues^36,37^, comparable to our results. For absolute quantification, our study showed substantial T1 overestimation and T2 underestimation for uninvolved masseter compared to literatures,^40^ consistent with bias in phantom. For tumor response, Bonate et al. reported significant longitudinal T2 increase over a cohort of 15 HNSCC patients treated with hypofractionated ART on a 1.5T MR-linac.^12^ In our analysis, within the base of tongue GTVs, T1 values showed marginal changes while 2 of those demonstrated more noticeable T2 enhancement (PostTx ΔT2_mean_>12.8%). The resolved significant nodal metastasis exhibited considerable decreases in QMRI endpoints instead (PostTx ΔT1_mean_=-39.5%, ΔT2_mean_=-32.7%). Within RD, histogram visualization better reflected the changes of pathology volume and composition while mean statistics suggested similar information as GTV. For the base of tongue RDs, T1 variations were minor while 2 of those also exhibited T2 enhancement (PostTx ΔT2_mean_>18.1%). The resolved significant nodal metastasis also demonstrated remarkable decreases in QMRI endpoints (PostTx ΔT1_mean_=-29.2%, ΔT2_mean_=-35.3%). For salivary gland response, Zhou et al. reported significant longitudinal increase of T2 values (6.0% MidTx and 4.6% PostTx-MidTx) in parotids analyzing 41 nasopharyngeal carcinoma patients.^41^ Literature is limited at assessing submandibular glands response using quantitative T1 and T2. In our study, for the first 2 patients who were imaged with the same coil at all timepoints, at PostTx, all involved parotids showed T1 increases (>12.4%) and 2 of those showed T2 increases (>13.4%), and all involved submandibular glands showed considerable T1 (>14.6%) and T2 (>34.5%) increases. Uninvolved parotid and submandibular gland remained relatively stable instead. The coincidence between substantial T1 and T2 changes in above salivary glands and both patients’ report of severe dry mouth indicates potential endpoints or predictors for functional adaptation.

DL-MUPA derives both T1 and T2 maps in a single rapid scan (4 min 30 sec for brain/6 min 30 sec for HnN) depending on FOV (192/264 mm^3^), acquisition resolution (1.5^3^/1.5×1.5×2 mm^3^) and averages (2/1.5). As a comparison, a previously reported STAGE method took 10 minutes on a 0.35T MR-linac to generate PD, T1 and R2* maps (232.5×310×192 mm^3^ FOV, 1×1×3 acquisition resolution, 12.5% oversampling) while the standard IR method can take a few hours for T1 mapping of a single slice.^14^ DL-MUPA also incorporates DL enhancement for noise and artifact reduction and sharpness improvement. Further acceleration can be achieved by reasonably reducing FOV, resolution and averages while increasing the power of DL enhancement, trade-off in image quality to be further assessed.

One limitation of our work is DL-MUPA was not benchmarked against established standard T1 and T2 mapping methods such as VFA and IR. As a surrogate, we benchmarked against manufacturer-provided reference in the phantom which yielded reasonable results.

Another limitation of our study was that during our longitudinal clinical studies spanning 28 months, the MR software was upgraded twice while the software versioning has shown to influence reconstruction algorithms and quantitative MRI.^42^ However, our one-year phantom benchmarking using the brain coil demonstrated excellent repeatability of DL-MUPA quantitative results despite different software versions. In addition, a new HnN coil was implemented during the course of study leading to use of different coils at different timepoints for two HnN patients in the analysis. Switching coils demonstrated potential impact on the quantitative analysis. Images especially DL-MUPA derived T2 maps showed different noise pattern, for instance in Fig 5 comparing PreTx with MidTx and PostTx. For this patient, T2 histograms of both submandibular glands showed similar pattern where MidTx and PostTx agreed well but both deviated from PreTx, while T1 histograms demonstrated different patterns. In addition, potential coil dependency of normal masseter T1 quantification was observed where T1 variations greater than 10.5% were found comparing timepoints acquired with different coils for the same subject. Nature of such discrepancies needs further validation in larger cohort. While prompt equipment evolvement was prioritized for patients’ benefit in our study, use of the same software and hardware in longitudinal studies can eliminate such confounding factors when conditions allow.

With overall excellent in-vivo repeatability, tumor change detectability and time efficiency are demonstrated, DL-MUPA shows promise in deriving QMRI biomarkers correlated to treatment response, to be validated in a larger cohort with clinical outcome information such as 2-year overall survival and normal tissue toxicities. Other potential applications of DL-MUPA include generating quantitative PD map from the PD-w images by local normalization after correcting B1 transmit and receive contributions, and generating synthetic CT from the Zero TE PD-w images to support MR-only radiation treatment workflow^19^.

## Conclusion

Our preliminary results demonstrate the feasibility of applying DL-MUPA for assessing longitudinal treatment response. DL-MUPA was stable in phantom and in uninvolved regions *in vivo*, suggesting excellent repeatability, while lesion and involved organs showed demonstrable changes highlighting the sensitivity of our technique. With further confirmation and coupled with clinical outcome information, further correlation with T1 and T2 via DL-MUPA can be established to further identify actionable endpoints for functional treatment adaptation.

## Supplementary Material

Supplement 1

## Figures and Tables

**Table 1.** Summarized results of 12-session phantom benchmarking, each session including 5 consecutive scans. T1 and T2 values within physiologically relevant range were reported. Results include mean derivations, intersession standard deviation (SD), intersession coefficient of variance (CV) evaluating the repeatability among the 12 sessions, range of intrasession CV evaluating the repeatability of the 5 consecutive acquisitions within a single session, and bias in absolute value and percentage comparing to manufacturer-provided reference values.

**T1 Quantification (N=12)**	**Vial**	**Mean (ms)**	**Intersession SD (ms)**	**Intersession CV (%)**	**Intrasession CV (%)**	**Reference (ms)**	**Bias (ms)**	**Bias (%)**
	1	2175.6	12.1	0.6%	[0.1%, 0.9%]	1741.3	434.3	24.9%
	2	1700.4	7.6	0.4%	[0.1%, 0.6%]	1269.8	430.6	33.9%
	3	1309.4	4.5	0.3%	[0.1%, 0.5%]	962.0	347.4	36.1%
	4	931.1	3.4	0.4%	[0.0%, 0.5%]	684.7	246.3	36.0%
	5	662.8	3.6	0.5%	[0.1%, 0.8%]	487.4	175.5	36.0%
	6	478.0	2.1	0.4%	[0.2%, 1.0%]	346.4	131.6	38.0%
	7	382.3	3.3	0.9%	[0.1%, 1.0%]	245.8	136.6	55.6%
**T2 Quantification (N=12)**	**Vial**	**Mean (ms)**	**Intersession SD (ms)**	**Intersession CV (%)**	**Intrasession CV (%)**	**Reference (ms)**	**Bias (ms)**	**Bias (%)**
	2	353.6	17.0	4.8%	[0.4%, 2.7%]	493.4	−139.8	−28.3%
	3	245.8	5.9	2.4%	[0.6%, 1.8%]	355.5	−109.7	−30.9%
	4	181.2	2.8	1.5%	[0.2%, 1.1%]	237.3	−56.1	−23.6%
	6	92.8	0.9	1.0%	[0.3%, 0.6%]	122.7	−29.9	−24.4%
	7	65.9	0.7	1.0%	[0.3%, 0.6%]	88.0	−22.1	−25.1%
	8	42.3	0.5	1.3%	[0.2%, 0.7%]	61.6	−19.4	−31.4%
	9	14.8	1.0	6.6%	[1.3%, 4.3%]	41.8	−27.0	−64.6%

**Table 2.** Longitudinal changes of mean T1 and T2 values and within-subject coefficient of variance (wCV) for each region of interest (ROI) for the two brain metastases patients.

**Patient A**	**ROI**	**4-mo PostTx ΔT1**_**mean**_ **(ms (%))**	**7-mo PostTx** **ΔT1**_**mean**_ **(ms (%))**	**T1** **wCV (%)**	**4-mo PostTx ΔT2**_**mean**_ **(ms (%))**	**7-mo PostTx ΔT2**_**mean**_ **(ms (%))**	**T2 wCV**

	**NAWM**	8 (1.1%)	20 (2.9%)	1.5	0 (0.9%)	0 (−0.6%)	0.8
	**GTV**	155 (13.7%)	193 (17.1%)	8.2	12 (17.8%)	11 (16.2%)	8.8

**Patient B**	**ROI**	**6-mo to 3-mo ΔT1**_**mean**_ **(ms (%))**	**T1 wCV (%)**	**6-mo to 3-mo ΔT2**_**mean**_ **(ms (%))**	**T2 wCV (%)**

	**NAWM**	36 (5.0%)	3.5	2 (5.0%)	3.4
	**GTV**	502 (39.7%)	23.4	41 (41.4%)	24.3
	**GTV 2**	214 (17.6%)	11.4	7 (8.7%)	5.9
	**GTV 3**	−52 (−4.6%)	3.4	−5 (−7.1%)	5.2

**Table 3.** Longitudinal changes of mean T1 and T2 values and within-subject coefficient of variance (wCV) for each region of interest (ROI) for the three head and neck (HnN) cancer patients. I-II had squamous cell cancer (SCC) of the right base of tongue, while III had SCC of the left based of tongue (GTV 1) and left nodal metastases (GTV 2)

Patient	ROI	MidTx ΔT1_mean_ (ms (%))	PostTx ΔT1_mean_ (ms (%))	T1 wCV (%)	MidTx ΔT2_mean_ (ms (%))	PostTx ΔT2_mean_ (ms (%))	T2 wCV (%)
**HnN Patient I**	**Masseter**	47 (5.0%)	18 (1.9%)	2.5	−2 (−5.6%)	−	3.4
	**GTV**	−76 (−6.5%)	−46 (−3.9%)	3.4	−1 (−1.9%)	7 (12.8%)	7.7
	**RD**	56 (4.8%)	66 (5.6%)	2.9	4 (6.9%)	10 (18.1%)	8.4
	**Parotid R**	12 (1.9%)	82 (12.4%)	6.4	−1 (−2.5%)	1 (2.4%)	2.5
	**Parotid L**	22 (3.3%)	38 (5.6%)	2.8	−2 (−4.6%)	−2 (−3.1%)	2.4
	**Submand Glnd R**	113 (12.3%)	135 (14.6%)	7.2	5 (10.8%)	17 (34.5%)	15.3
	**Submand Glnd L**	30 (3.2%)	69 (7.3%)	3.5	−2 (−3.9%)	3 (6.3%)	5.1

**HnN Patient II**	**Masseter**	22 (2.4%)	53 (5.7%)	2.8	1 (4.5%)	4 (11.8%)	5.6
	**GTV**	13 (1.1%)	−23 (−1.8%)	1.5	−1 (−2.5%)	1 (2.1%)	2.3
	**RD**	26 (2.1%)	−3 (−0.3%)	1.3	0 (0.3%)	−2 (−4.4%)	2.7
	**Parotid R**	71 (10.2%)	185 (26.6%)	12.0	4 (8.9%)	8 (18.4%)	8.4
	**Parotid L**	50 (7.0%)	157 (22.3%)	10.4	2 (4.6%)	6 (13.4%)	6.4
	**Submand Glnd R**	241 (28.1%)	376 (43.8%)	17.9	15 (33.9%)	26 (61.3%)	23.3
	**Submand Glnd L**	150 (17.9%)	242 (28.9%)	12.6	7 (15.4%)	16 (36.4%)	15.6

**HnN Patient III**	**Masseter**	−142 (−12.6%)	−118 (−10.5%)	7.3	1 (6.7%)	2 (8.0%)	4.1
	**GTV 1**	−21 (−1.8%)	90 (7.6%)	4.9	2 (4.6%)	15 (33.4%)	16.1
	**RD 1**	15 (1.3%)	117 (9.9%)	5.2	4 (8.3%)	16 (36.6%)	16.7
	**GTV 2**	38 (2.8%)	−541 (−39.5%)	26.9	0 (0.5%)	−24 (−32.7%)	21.3
	**RD 2**	82 (6.0%)	−400 (−29.2%)	20.4	3 (3.6%)	−25 (−35.3%)	24.0
	**Parotid R**	−30 (−4.3%)	23 (3.3%)	3.8	2 (3.3%)	2 (3.3%)	1.9
	**Parotid L**	−29 (−4.0%)	24 (3.3%)	3.6	−2 (−4.0%)	0 (0.1%)	2.4
	**Submand Glnd R**	−64 (−8.1%)	10 (1.2%)	5.2	2 (5.4%)	7 (15.3%)	7.3
	**Submand Glnd L**	−7 (−0.9%)	117 (15.1%)	8.6	8 (18.7%)	10 (23.2%)	10.8

## Data Availability

Research data are not available at this time.
