# Supplementary material for: Evaluation of a Novel Quantitative Multiparametric MR Sequence for Radiation Therapy Treatment Response Assessment
Source: ArXiv. 2025 Mar 28:arXiv:2503.22640v1. Preprint. [Version 1] (PMC11975303)
Supplement: Supplement 1 [file NIHPP2503.22640v1-supplement-1.pdf]

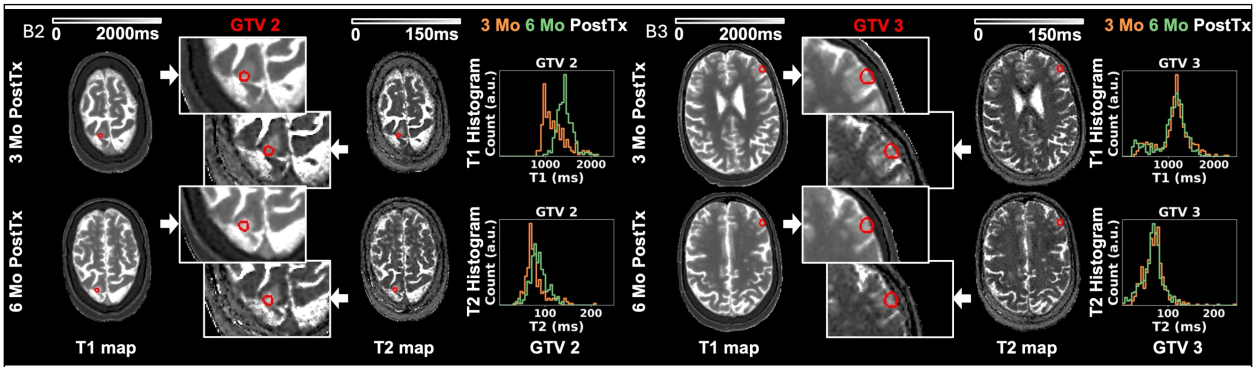

Appendix Figure S1. Post-contrast T1 Stealth and T2 FLAIR, longitudinal DL-MUPA derived T1 and T2 maps (global and magnified to metastases), and T1 and T2 histograms for the brain metastases patient B in figure 2. The right precentral gyrus lesion (GTV2) exhibited considerable T1 and T2 enhancement compared to 3-month PostTx while the left middle frontal gyrus one (GTV3) remained stable.
